# NQR Characteristics of an RDX Plastic Explosives Simulant

**DOI:** 10.1007/s00723-012-0337-6

**Published:** 2012-05-03

**Authors:** J. Turecek, B. Schwitter, D. Miljak, M. Stancl

**Affiliations:** 1 Police Academy of the Czech Republic, Prague, Czech Republic; 2 Minerals Down Under National Research Flagship, Commonwealth Scientific and Industrial Research Organisation (CSIRO), Kirrawee, Australia; 3 Division of Process Science and Engineering, Commonwealth Scientific and Industrial Research Organisation (CSIRO), Kirrawee, Australia; 4 Explosia a.s, Pardubice, Czech Republic

## Abstract

For reliable detection of explosives, a combination of methods integrated within a single measurement platform may increase detection performance. However, the efficient field testing of such measurement platforms requires the use of inexplosive simulants that are detectable by a wide range of methods. Physical parameters such as simulant density, elemental composition and crystalline structure must closely match those of the target explosive. The highly discriminating bulk detection characteristics of nuclear quadrupole resonance (NQR) especially constrain simulant design. This paper describes the development of an inexplosive RDX simulant suited to a wide range of measurement methods, including NQR. Measurements are presented that confirm an RDX NQR response from the simulant. The potential use of the simulant for field testing a prototype handheld NQR-based RDX detector is analyzed. Only modest changes in prototype operation during field testing would be required to account for the use of simulant rather than real explosive.

## Introduction

The detection of explosives during security checks of persons, luggage, consignments, vehicles, etc. is a very difficult task. This is especially true in the case of technically masked improvised explosive devices. A combination of physical methods may be necessary for reliable detection. Testing of instruments for correct system functionality and sensitivity may therefore require material compatible with detection by multiple methods. The response characteristics of the material must also allow for relevant detection scenarios useful for training security personnel. However, in many cases it is not desirable or even possible to use real explosives for testing. Instead, an inert material must be used to simulate the actual physical characteristics of an explosive material that are crucial for detection and identification.

Certain kinds of simulants are used to test security systems, but for some methods of detection there are no suitable simulants available. Moreover, each of the existing simulants are either specific only for one method, or at a maximum for one small group of detection methods. They include inert materials for visual and tactile recognition, X-ray methods involving dual energy [backscatter and computer tomography (CT)], nuclear methods such as neutron activation of gamma rays and samples of substances for trace particles detection with devices or dogs. Research on an RDX simulant for nuclear quadrupole resonance (NQR) has also been previously conducted [1]. However, for advanced measurement platforms in real security testing applications, a simulant has to satisfy the requirements of a combination of detection methods based on different physical principles. Therefore, it is necessary to develop substances that cannot explode and simultaneously simulate real explosives with the maximum number of physical characteristics crucial for their detection. The development of a universal simulant is particularly complicated with regard to bulk detection methods where the signal depends on the chemical bonds in the search target explosive. Such methods include X-ray diffraction, NQR and nuclear magnetic resonance (NMR). These methods in principle have very small probability of false positive alarms, but only NQR seems suitable, for practical reasons, to search for plastic explosives on persons. It is therefore of special interest to explore the NQR characteristics of a universal RDX plastic explosive simulant.

## Methods used in the Detection of Explosives

In the first phase of the research, it was necessary to carry out a detailed theoretical analysis of the physical interactions underlying the bulk and trace methods of explosives detection. The following section briefly describes the relevant physical parameters that impact on simulant design, for methods currently exploited in existing devices or proposed methods for checking of persons, luggage, consignments and vehicles.

### X-Ray Methods

X-ray machines are the most widely deployed devices for security checking. Besides simple absorption of X-ray radiation, the devices also employ the methods of backscattering (Compton scattering), dual (multi) energy (of X-ray photons), CT and X-ray diffraction. At airports, there are automatic explosive detection systems based on the last three physical principles.

X-ray machines with dual (multi) energy scan two (more) X-ray images of the controlled object from the same angle, but for different wavelengths of X-ray radiation. They use the fact that the coefficient of absorption also depends on the wavelength of X-ray radiation. The intensity of X-ray radiation of the first wavelength upon transmission through a controlled object with thickness *d* is $I_{ 1} = I_{0 1} e^{{ - {{\upmu}}_{1} \cdot d}}$, where *I*_01_ is the initial intensity and μ_1_ the attenuation coefficient. Similarly for the second wavelength, $I_{ 2} = \, I_{0 2} e^{{ - {{\upmu}}_{2} \cdot d}}$. Therefore, μ_1_/μ_2_ = ln (*I*_01_/*I*_1_)/ln (*I*_02_/*I*_2_), and it is evident that there is no dependence on the thickness of the material of the controlled object. Furthermore, the ratio μ_1_/μ_2_ depends on the effective proton number *Z*_eff_ of the material of the controlled object. The essential relevant point for simulant design is that *Z*_eff_ is approximately proportional to the product of density and average proton number *Z*. It means that the simulant should have similar density and average proton number as the original explosive.

Security X-ray machines with CT scan X-ray images of the object from different directions and these images are then mutually computed. On the basis of this comparison, the absorption of X-ray radiation in any elementary region of the examined object can be determined. The region may be assigned a material mass density. Explosives are then detected simply on the basis of their CT density. Therefore, the simulant should have similar density and average proton number as the original explosive.

X-ray diffraction (XRD) imposes different constraints on simulant design. In X-ray diffraction, an incident X-ray of a particular wavelength is diffracted by the crystalline phase of the specimen according to Bragg’s law. A given crystal structure corresponds to a distinctive diffraction pattern. For some crystalline materials, it is sometimes possible to obtain isomorphic structures (similar crystalline lattice parameters but with different atom scatterers, as occurs in some mineral families with different levels of atom substitution). Such isomorphic structures may result in similarities in the main diffraction peaks. However, crystalline explosives would seem unlikely to allow this possibility because of the difficulty in finding suitable atom substitutions. Therefore, for XRD detection the simulant should have a significant content of RDX.

### Neutron Methods

In recent years, there has been promising development of methods using neutron radiation for explosives detection. The simplest of them, neutron backscattering, involves irradiating the controlled object by a beam of fast neutrons followed by thermalized neutron detection, i.e., the neutrons slowed down by the nuclei of hydrogen or other light atoms. More advanced methods include neutron in–gamma out methods such as thermal neutron analysis (TNA), fast neutron analysis (FNA), pulsed fast neutron analysis (PFNA), pulsed fast-thermal neutron analysis (PFTNA) and nanosecond neutron analysis/associated particles imaging (NNA/API). These methods are based on the irradiation of a controlled object (luggage) by a beam of neutrons with the subsequent detection of gamma radiation as a product of the interaction of neutrons with nuclei of chemical elements contained in the controlled object. Each chemical element produces characteristic gamma radiation. Considering these neutron methods, the simulant will present as the original explosive as long as it contains basic elements, i.e., carbon, oxygen, nitrogen and hydrogen, with the same *mutual ratio* as in the actual explosive.

There also exist systems exploiting the simultaneous measurement of transmitted fast neutrons and X-rays [2] through material. These are examples of an advanced detection system based on two different underlying methods. In this case, the simulant must satisfy the constraints imposed by both X-ray and neutron measurement mentioned above.

### Trace Particle Detection

Currently, trace particle detectors are widely deployed for security checking of persons, luggage, consignments or vehicles. These detectors take samples by vacuuming vapors from the vicinity of the controlled object or by wiping its surface. The analysis process itself may involve ion mobility spectroscopy (IMS), various combinations of (dual) gas chromatography (DGC), preselection by a semipermeable membrane, various preconcentrations on special surfaces, electron capture detection (ECD), mass spectrometry (MS) or biodetection, etc. The devices are not configured to detect inexplosive components of explosives such as binders, dyes, etc.^1^ They always detect the presence of the energetic components of explosives. Based on these considerations, the inert simulant must contain at least a small amount of the explosive component if it is to present as the original explosive. Regarding trace particle detection by dogs, the substances that are included in the simulant should not have a strong smell [3].

### Millimeter Wave Imaging

Passive and especially active millimeter wave imaging systems are used for body scanners. These methods rely on measuring changes in millimeter wave reflectivity due to variations in dielectric constant and electromagnetic loss tangent of the material. Contrast in images may be obtained between human skin and other objects because of differences in the electromagnetic parameters. Very high resolution imaging through thin dielectric layers like clothing is possible. Many liquids and lossy materials provide high contrast. However, the selectivity for two different types of materials with similar electromagnetic parameters may be limited. Simulant characteristics relating to this measurement method were not considered explicitly in this study. However, it is reasonable to assume that a bulk volume of simulant material will behave similarly to the corresponding explosive. This is because explosives, binders and other materials potentially suited to simulant preparation have broadly similar dielectric characteristics (relatively low dielectric constant and loss tangent).

### Nuclear Quadrupole Resonance

NQR involves detection of spin resonances in the radiofrequency spectral range. The resonant frequencies are highly dependent on the electronic coordination around a given nucleus. For ^14^N nuclei in explosives, the resonances generally occur below 6MHz, with spectral widths of tens of hertz up to a few kilohertz [4, 5]. It may be argued that for explosives detection, the NQR method yields possibly the highest specificity of all considered methods. Just like the XRD method, it is difficult to replicate the NQR response using other substances. Therefore, the simulant should have a sufficient content of RDX with regard to NQR measurement. What is more, the preparation method must not lead to excessive broadening of the resonance due to even subtle changes in the electric field gradient distribution in the vicinity of the target nuclei.

NMR has also been proposed for explosives detection [6]. The measurement of characteristic ^1^H NMR decay times in liquid or crystalline solid explosives may facilitate specific detection. NMR decay times in liquids are generally much longer than in solids. It is therefore unlikely that a solid simulant developed for NQR would be suitable for testing NMR methods configured for liquid explosive detection. However for the case of NMR measurement of solid explosives, a simulant developed for NQR would be suitable to the extent of providing a significant NMR response with exactly the same decay characteristics as the actual explosive. The ^1^H response from the inert materials in the simulant may also contribute response components with short decay times.

## The Proposal for an Inert Simulant

On the basis of the analysis of underlying physical interactions used for explosives detection, the individual methods were grouped into two sets according to the demands made on the composition of the simulant (see Tables 1, 2). Table 1 includes cases where required physical parameters may be met by the use of inert material only, while Table 2 includes cases where the energetic material (original explosive) must be mixed with the inert material.

**Table 1:** Group of explosives detection methods which require a simulant realized using a basic inert mass only

Group of explosives detection methods	Required physical characteristics
X-ray absorption and backscattering (simple absorption, backscattering—Compton scattering, automatic explosives detection by double (multi) energy (from one and more angles of view), CT computer tomography)	Corresponding effective proton number It would be also met with the corresponding proportional representation of elements (N, O, C, H)
TNA—Thermalized neutron detection	Higher amount of hydrogen atoms
Neutron in–gamma out methods (TNA—thermal neutron analysis, FNA—fast neutron analysis, PFNA—pulsed fast neutron analysis, NNA/API—nanosecond neutron analysis/associated particle interrogation)	Corresponding proportional representation of elements (N, O, C, H)

**Table 2:** Group of explosives detection methods which require a simulant realized by a mixture of basic inert mass with an inexplosive concentration of RDX component

Group of explosives detection methods	Required physical characteristics
Trace particles detection (IMS—ion mobility spectrometry, GC—gas chromatography, ECD—electron capture detection, MS—mass spectrometry, etc.)	Presence of a small amount of the original explosive component (RDX in this case)
Optical standoff methods (Raman spectrometry, terahertz spectrometry, LIBS—laser induced breakdown spectrometry, etc.)	Presence of a detectable amount of the original explosive component (RDX)
Magnetic resonance (NQR, NMR)	Presence of a larger (detectable) amount of the original explosive component (RDX)
X-ray diffraction (XRD)	Presence of a larger (detectable) amount of the original explosive component (RDX)

Most of these requirements were realizable within an adequate accuracy if the simulants were prepared according to the following procedure: firstly, to propose a composition of basic inert mass with proportional content of basic elements N, O, C and H that correspond most favorably to the original explosive. Then, after verifying the required characteristics of the basic inert mass, experiments were performed to confirm the maximum inexplosive concentration of RDX to be mixed with the basic inert mass.

Several tens of organic compounds were considered for the preparation of samples. Especially, oxalic acid and its salts appeared suitable for this purpose. Unfortunately, these compounds are chemically aggressive or, from the physical point of view, are absolutely incompatible with bonding systems of plastic explosives. A mixture of cyanuric acid, tetrazole (guanidine azo), CMC (carboxy methyl cellulose), water and glycol have been found suitable.

The maximum guaranteed inexplosive concentration of RDX in the mixture with basic inert mass was experimentally stated at 20 %. The Czech certified Safety Engineering Laboratory of the Research Institute for Industrial Chemistry, Explosia a.s. performed sensitivity tests according to the international certified procedures [3]. The tests included mechanical sensitivity with respect to shock, the test of mechanical sensitivity with respect to friction and the test of thermal sensitivity. The simulant was not sensitive in any case. In terms of safe international transportation, the Czech Proof House for Arms and Ammunition classified the simulant as 4.1 material (flammable solids, self-reactive substances and solid desensitized explosives). The 20 % RDX content is actually about the maximum limit also because the other components of the universal simulant must have the same ratio of chemical elements N, O, C and H like the real plastic explosive. This constraint requires a higher oxygen content and places a severe limitation on the search for binder material with better desensitizing properties.

The comparison of the density and N, O, C, H elemental composition for the basic inert mass, the final simulant and actual RDX-based explosive is presented in Table 3. The concentrations of C, H, N and O were measured by an automatic elemental analyzer (EA 1108 Fisons at Elemental Analysis Service, Department of Organic Chemistry, Faculty of Chemical Technology, The University of Pardubice).

**Table 3:** Comparison of density and N, O, C and H elemental composition for the basic inert mass, the final simulant and original explosives of the hexogen type

	Density (g cm^−3^)	N (mass %)	O (mass %)	C (mass %)	H (mass %)
Basic inert mass	1.51	30.4	40.1	24.5	5.0
Final simulant (20 % RDX)	~1.43	32.09	38.40	25.42	4.09
C-4^a^ explosive Pl SE M	1.53	30.26 29.88	41.71 39.73	24.35 26.38	3.68 4.01

Regarding the elemental composition ratios, it should be noted that different types of RDX-based explosives have variations in composition, according to manufacturing process. There is therefore no exact prescription for the elemental ratios and densities required of a simulant. Rather, common RDX-based explosives have elemental ratios that fall within a relatively narrow range of concentrations. It is sufficient if the measured difference between the simulant and the original explosives is similar to the difference between the common RDX based explosives themselves. Moreover, the detection of explosives is complicated in practice by the presence of materials surrounding the controlled object. It is therefore unlikely that small variations in simulant elemental composition compared to a given RDX-based explosive target would lead to significant changes in detection efficiency for equipment under test.

As a first step in evaluating the detection characteristics of the simulant, a sample of the simulant was submitted for NQR measurement. As discussed above, the NQR response is probably the most difficult to mimic in a simulant. The aim of the measurements was to firstly confirm detection of a useable NQR response from the simulant material, and secondly to consider limitations imposed by differences in sensitivity between the simulant and actual explosives. These measurements are described in the next section.

## Simulant NQR Measurements

The CSIRO is developing a prototype handheld probe for personnel scanning based on NQR [7]. For comprehensive characterization of system performance, the probe needs to be tested in real-world environments on actual human subjects under a variety of conditions. Due to health and safety concerns, the carriage and handling procedures for explosives are restrictive, and this may limit the scope of testing. A suitable simulant would provide the convenience of relaxing the operational constraints associated with field testing.

The NQR measurement poses a difficulty for simulant preparation because of the high discrimination of the technique afforded by narrow radiofrequency resonances, together with the fact the method requires bulk mass for detection. For a given ^14^N NQR, target spectral interference due to other materials is usually rare. The available magnetic resonance literature suggests it is difficult to find two substances having closely matched resonant frequencies and spin decay times. Previously, CSIRO has used the 3.6 or 4.64 MHz lines in sodium nitrite (NaNO_2_) [8] as a substitute for RDX resonances. However, from a systems testing point of view, the frequency difference of the two materials is significant enough to be problematic for extrapolation of system performance to the RDX resonant frequency (5.192 MHz). This is because most NQR systems are configured for narrow band transmission and detection. Also different magnetization decay times lead to the requirement for differing pulse sequences for optimum performance for each material. Sodium nitrite has the additional property of strong piezoelectric response, and we have found that mechanical treatment of some sodium nitrite samples (shaking of samples and thermal shocks, even for samples potted in paraffin wax or finely ground) can cause noticeable changes in response. The use of a simulant containing RDX would remove uncertainties in systems testing. It would also be of benefit if the simulant were suitable for other methods besides NQR, especially for cases where multiple methods are assessed simultaneously, either separately or as part of multi-sensor integrated systems.

To characterize the RDX, simulant response measurements were compared to two other samples containing RDX: (i) a sample of PE4 with nominal RDX content of 87 %, phlegmatized with lithium stearate grease, paraffin and pentaerythrytol dioleate and (ii) a sample of Primasheet^®^ 2000, a flexible sheet explosive (3 mm thickness) composed of 88 % RDX (nominal), with the remainder mostly composed of polyisobutylene, other plasticisers (sebecates/adipates) and Teflon^®^. Each of the PE4 and simulant (100.0 g samples) were pressed by hand into the shape of a disc at the bottom of identical cylindrical plastic containers. The resulting discs had thicknesses of approximately 1 cm and diameter 9.5 cm. It was found during this process that the simulant was less malleable than PE4. For the Primasheet^®^ sample, two oblong sections of sheet, each with edge lengths 9.5 × 11 cm, were stacked on top of each other. The total Primasheet^®^ mass was 106.0 g.

The measurements were performed using the CSIRO prototype handheld NQR personnel scanning prototype, configured for the detection of the RDX ^14^N resonance at 5.192 MHz. The prototype consists of a coil set arranged to provide a high level of radio frequency interference suppression. The suppression level is such that in typical open environments, the total NQR receiver noise is dominated by the thermal noise in the coil set. The measurements reported here were performed in an unshielded environment. The active aperture of the coil set is a circle with diameter approximately 10 cm. Therefore, the differences in the sample spatial distribution between Primasheet^®^ and the other plasticised RDX samples described above should not have a large effect on the relative responses.

**Fig. 1:**
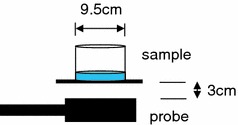
Schematic of the measurement geometry

To determine response parameters of each sample at high signal to noise ratio, the samples were fixed in a position adjacent to the active probe aperture at a standoff distance of 3 cm. Figure 1 shows the measurement configuration. In addition, the dwell time for measurement was extended considerably compared to normal prototype operation, so as to allow the averaging of 4,096 waveforms. The pulse width was 150 μs, with a 450 μs delay applied between the end of the pulse and the beginning of data acquisition. The pulse repetition period τ was 64 ms, long enough to allow magnetization recovery and decouple the response of successive pulses, i.e., no steady state or echo component is expected, since *T*_1_ (the spin–lattice relaxation time) is significantly shorter than the pulse repetition period. This was confirmed by noting that no change occurred in the observed amplitudes after increasing the pulse repetition rate by a factor of two.

**Fig. 2:**
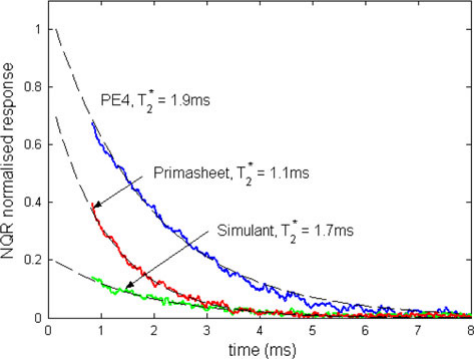
Free induction decay magnitudes for the three samples, normalized to that of the PE4 sample. Extrapolation of curves back to *t* = 0.15 ms yields the relative response

Figure 2 shows the free induction decay magnitude for the three tested samples, normalized to the case of PE4. Exponential fits to the free induction decay are also shown that yield the inverse linewidth parameter *T*_2_*. The PE4 has the longest decay time (*T*_2_* = 1.9 ms), Primasheet^®^ the shortest (*T*_2_* = 1.1 ms), while the simulant has an intermediate value (*T*_2_* = 1.7 ms). Extrapolation of the exponential decays to the time at the end of the excitation pulse provides an estimate of the relative response of each sample. Since PE4 and simulant samples have identical presentation geometries, their responses are directly comparable. The simulant has about 20 % of the response of PE4. This is entirely consistent with the known concentrations of RDX in each sample, assuming that the intrinsic response of the entrained RDX in each sample is similar. The lower response of the Primasheet^®^ sample compared to the PE4, despite very similar nominal RDX content, may be related either to intrinsic variation or to differences in the sample presentation.

A further measurement involved testing for relative *T*_1_ between the samples. This was achieved for each sample by comparing the reduction in signal over a range of τ starting from τ = 128 ms down to τ = 16 ms. The relative reduction in signal observed for each sample was the same to within the measurement reproducibility, thus indicating the same *T*_1_ value for each sample. In addition, the chosen NQR transition frequency for each sample was found to be the same within a few hundred Hertz, which is the resolution limit imposed by the temperature control in the experiment (~0.5 K) and the known temperature coefficient [9] for this resonance (~500 Hz/K). It is likely that the spin dynamics of RDX in each sample is very similar, but the differences in inhomogeneous broadening observed are related to the particular crystalline quality imparted by the method of RDX entrainment.

Finally, limitations due to the reduction in response for the simulant should be considered from an operational point of view. For example, previous initial work has demonstrated that a maximum handheld probe scanning head speed of 1.5 m/s is compatible with detection of a 100 g PE4 sample (with similar geometry to the samples used in this work) situated 5 cm from the probe head. The factor of 5 reduction in simulant response implies that a factor of 25 increase in dwell time of the probe head over the sample is required, or an equivalent maximum scan speed of 0.06 m/s. This is too slow to provide useful real-world testing. However, initial testing suggests that a closer spacing between the probe head and personnel is possible (approximately 3 cm). This increases the probe head maximum speed by a factor of about 5–10, depending on the particular pulse scheme. If it is also practical to increase the simulant sample volume by a factor of 2–3 compared with the standard volume of PE4; then, this would allow for rapid probe head scanning speeds in test work involving simulants. It may therefore be concluded that the simulant will serve as a safe and suitable substitute for real explosive during NQR system testing, within the modest restrictions discussed.

It is worthwhile noting that the test work restrictions described above would not arise in the testing of most other detection methods, where it is expected that comparable sensitivity to actual explosives would be recovered. Simulants with higher content of RDX, which would be an advantage to NQR system testing, can also be prepared if it is assumed that desensitizing is the only requirement of the added inert materials. However, such simulants would not be suitable for testing other detection systems, such as X-ray based systems, which are commonly deployed in existing security checkpoints. Only simulants of the type described in this paper would be suited to testing systems that employ methods like NQR in parallel with the common X-ray methods.

## Conclusion

On the basis of an analysis of a number of explosive detection principles, an RDX simulant has been prepared that meets the requirements for a wide range of detection methods. The N, O, C and H elemental composition has been closely matched to common RDX-based explosives. Moreover, the 20 % RDX content in the simulant allows for testing methods such as NQR that have very high specificity according to electronic coordination. The simulant has been certified as an inexplosive material. NQR measurements on the simulant demonstrate the existence of a suitable response with decay characteristics similar to other RDX-based explosives. The potential use of the simulant for testing a prototype handheld NQR-based probe has been analyzed. The reduction in NQR sensitivity provided by the simulant compared to actual explosive material is expected to lead to only modest changes in the operational testing regime for the prototype. The NQR method imposes the most stringent limitation in this regard, and it is expected that other methods would recover normal sensitivity from the simulant in field test work.

## Footnotes

1 The devices are not configured to detect inexplosive components of explosives except for detection of 2,3-dimethyl-2,3-dinitrobutane (DMNB) and *para*-mononitritoluene (*p*-MNT). The DMNB and *p*-MNT are marking agents specified in the Technical Annex of Montreal Convention on the Marking of Plastic Explosives for the Purpose of Detection.
